# Discovery of a novel lipid metabolism-related gene signature to predict outcomes and the tumor immune microenvironment in gastric cancer by integrated analysis of single-cell and bulk RNA sequencing

**DOI:** 10.1186/s12944-023-01977-y

**Published:** 2023-12-02

**Authors:** Jinze Zhang, He Wang, Yu Tian, Tianfeng Li, Wei Zhang, Li Ma, Xiangjuan Chen, Yushan Wei

**Affiliations:** 1 Department of Epidemiology and Health Statistics, School of Public Health, Dalian Medical University, Dalian, China; 2 Department of Scientific Research, First Affiliated Hospital of Dalian Medical University, Dalian, China; 3 Department of General Surgery, Institute of Precision Diagnosis and Treatment of Gastrointestinal Tumors, Shenzhen University General Hospital, Shenzhen University, Shenzhen, China; 4 Affiliated Shenzhen Maternity and Child Healthcare Hospital, Southern Medical University, Shenzhen, China; 5 Department of Obstetrics, Shenzhen University General Hospital, Shenzhen University, Shenzhen, China

**Keywords:** Lipid metabolism-related gene, Prognostic model, Gastric cancer, Single-cell RNA sequencing, Tumor immune microenvironment

## Abstract

Gastric cancer (GC) is a pressing global clinical issue, with few treatment options and a poor prognosis. The onset and spread of stomach cancer are significantly influenced by changes in lipid metabolism-related pathways. This study aimed to discover a predictive signature for GC using lipid metabolism-related genes (LMRGs) and examine its correlation with the tumor immune microenvironment (TIME). Transcriptome data and clinical information from patients with GC were collected from the TCGA and GEO databases. Data from GC samples were analyzed using both bulk RNA-seq and single-cell sequencing of RNA (scRNA-seq). To identify survival-related differentially expressed LMRGs (DE-LMRGs), differential expression and prognosis studies were carried out. We built a predictive signature using LASSO regression and tested it on the TCGA and GSE84437 datasets. In addition, the correlation of the prognostic signature with the TIME was comprehensively analyzed. In this study, we identified 258 DE-LMRGs in GC and further screened seven survival-related DE-LMRGs. The results of scRNA-seq identified 688 differentially expressed genes (DEGs) between the three branches. Two critical genes (GPX3 and NNMT) were identified using the above two gene groups. In addition, a predictive risk score that relies on GPX3 and NNMT was developed. Survival studies in both the TCGA and GEO datasets revealed that patients categorized to be at low danger had a significantly greater prognosis than those identified to be at high danger. Additionally, by employing calibration plots based on TCGA data, the study demonstrated the substantial predictive capacity of a prognostic nomogram, which incorporated a risk score along with various clinical factors. Within the high-risk group, there was a noticeable abundance of active natural killer (NK) cells, quiescent monocytes, macrophages, mast cells, and activated CD4 + T cells. In summary, a two-gene signature and a predictive nomogram have been developed, offering accurate prognostic predictions for general survival in GC patients. These findings have the potential to assist healthcare professionals in making informed medical decisions and providing personalized treatment approaches.

## Introduction

Globally, gastric cancer (GC) is the third most frequent cancer and the fifth most common cause of cancer-related death [1]. GC is widely distributed globally, but its incidence varies significantly in different regions. High-risk areas include East Asia (such as Japan, South Korea, and certain regions of China), Eastern Europe, and South America [2, 3]. In North America and Western Europe, there are fewer cases of stomach cancer. The prevalence of stomach cancer increases with age, especially among the middle-aged and elderly population, particularly those aged 50 and older [4]. However, lately, there have been reports demonstrating a rise in the frequency of stomach cancer among younger people. Despite significant advancements in clinical practices for the identification and management of GC, the persistent hurdles of insufficient early detection and limited treatment options for advanced cases have contributed to a 5-year survival rate of less than 10% [5, 6]. This is still the case despite substantial advancements in the clinical diagnosis and management of GC. Thus, the identification and evaluation of tumor prognostic indicators are of critical importance for the evaluation of tumor progression, the prediction of therapy efficacy, the reduction of the recurrence rate and mortality, and the extension of survival time.

Lipid metabolism refers to the synthesis, breakdown, and utilization processes of fats in the body [7]. Fats are essential nutrients and include fatty acids, glycerol, and some fat-soluble vitamins. Lipid metabolism plays a crucial role in various essential functions within the human body [8]. First, fats serve as a vital energy source. Whenever the body demands energy, lipids undergo decomposition into fatty acids and glycerol, subsequently undergoing further processing to generate ATP, the primary fuel for powering physiological functions [9, 10]. Second, fats play a crucial role in regulating body temperature, as they provide insulation when stored in subcutaneous tissue, reducing heat loss [11]. Additionally, fats are essential components in the construction of cell membranes, and the structure and function of cell membranes depend on the synthesis and regulation of fats [12]. Furthermore, fats are involved in the synthesis of hormones and biologically active substances such as vitamin D. Many studies indicate that changes in lipid metabolism could have an essential part in tumor formation and progression [13, 14]. According to certain research, tumor cells demonstrate considerable changes in lipid metabolism. To meet the energetic and metabolic requirements of their rapid expansion, tumor cells frequently display enhanced fatty acid production and esterification pathways. This aberrant lipid metabolism may enable tumor cells to grow, invade, and spread [15, 16]. On the other hand, research has also shown that lipid metabolism abnormalities can influence the host's immune response to tumors. For instance, lipid metabolism disturbances may lead to alterations in immune cell function, thereby weakening the host's antitumor immune response [17, 18]. Additionally, lipid metabolism abnormalities can impact the formation of the tumor microenvironment, creating a more favorable environment for tumor growth and invasion. Furthermore, there is an association between certain inherited lipid metabolism disorders and tumor development. For example, certain genetic mutations related to lipid metabolism, such as mutations in genes associated with triglyceride metabolism, have been found to increase the risk of specific types of tumors [19, 20]. Aberrations in lipid metabolism have been linked with a heightened susceptibility to GC according to numerous studies. For instance, stomach cancer has been associated with elevated cholesterol levels. Additionally, lipid metabolism abnormalities may also impact the prognosis of GC. According to research findings, specific markers associated with lipid metabolism have shown potential associations with the aggressive nature and poor prognosis of GC [21, 22]. In recent years, research has revealed significant new insights into the role of lipid metabolism in GC. Lipid metabolism plays a key role in cancer cell proliferation, particularly through pathways such as fatty acid synthesis. Additionally, it impacts the tumor microenvironment by controlling processes such as inflammation, immune suppression, and angiogenesis, which are vital for GC development. Specific lipid molecules, including phosphatidylinositol, fatty acids, and cholesterol metabolites, also regulate signaling pathways linked to tumor growth, invasion, and metastasis. Furthermore, the involvement of lipid metabolism in drug resistance poses a significant challenge in treating GC [23–25]. It was reported that reprogrammed lipid metabolism allows GC cells to use lipids as an energy source, supporting their rapid growth and division. This metabolic adaptation helps tumor cells thrive in the challenging tumor microenvironment. Lipid metabolism reprogramming may also facilitate the spread (metastasis) of GC to other parts of the body. Lipids can provide the necessary building blocks for the formation of new membranes in migrating cancer cells. In addition, abnormal lipid metabolism can contribute to resistance to various cancer treatments, including chemotherapy and targeted therapies. GC cells may develop mechanisms to evade the effects of these therapies by altering their lipid metabolism [21, 23]. An applicable technique for evaluating prognostic performance was to construct a model of the risks associated with prognosis. Various risk models have been created to investigate the prognostic significance of genes linked to the tumor microenvironment, immune cell infiltration, and energy metabolism in GC. However, lipid metabolism-related genes (LMRGs) in GC remain incompletely understood.

Single-cell RNA sequencing (scRNA-seq) is a high-throughput analysis technique used to sequence the genome, transcriptome, or epigenome of individual cells [26]. Traditional sequencing methods typically require a large number of cells, which are mixed together for sequencing, thereby masking the individual differences between cells [27]. In contrast, single-cell sequencing technology allows sequencing at the level of single cells, enabling researchers to understand and compare the genetic information and functions of different cells [28, 29]. The development of single-cell sequencing technology provides researchers with detailed insights into cells, revealing the heterogeneity of cells in development, disease, and other biological processes. Through single-cell sequencing, different cell types, subtypes, and transitions in cell states can be identified. Additionally, single-cell sequencing can uncover interactions and communication networks between cells. It is important to note that the diverse composition of immune cell infiltrates is a critical component in determining the therapeutic response and prognosis in GC as well as in other types of tumors [30, 31]. Unfortunately, because scRNA-seq requires a significant financial investment, only a few sample datasets were selected. Regarding the analysis of characteristics pertaining to individual cell subpopulations derived from bulk samples and the interplay between each cell and the TIME, the information garnered through single-cell RNA sequencing (scRNA-seq) holds significant potential for utility.

The purpose of this research was to employ single-cell sequencing and transcriptome sequencing to create a novel risk signature based on LMRGs that may be used for risk assessment and clinical decision making in GC. Furthermore, our findings could open up new avenues for drug development and targeted therapies that specifically address the dysregulation of lipid metabolism in GC. Ultimately, we aspire to contribute to improved patient outcomes and a deeper understanding of the molecular intricacies underlying this complex disease.

## Materials and methods

### Data collection

To locate genes involved in lipid metabolism (LMRGs), the MSigDB "Lipid Metabolism" category was examined. From The Cancer Genome Atlas (TCGA) database, 375 GC specimens and 32 surrounding normal specimens were extracted for high-quality mRNA analysis. Additionally, clinical information, such as grade, age, sex, pathological stage, N stage, T stage, and M stage information, was retrieved from the TCGA databases. Using the Perl programming language, the data were compared between gene expression profiles and clinical data, with any ambiguous or missing clinical data being eliminated. Subsequently, the R programming language was utilized to investigate the relationship between survival status and gene expression. Furthermore, we analyzed data from a total of 707 tumor samples, along with their corresponding normal cells, which were sourced from the Gene Expression Omnibus (GEO) database under accession number GSE112302. Additionally, we incorporated RNA-seq data from 434 GC samples from the GSE84437 dataset and 300 GC samples from GSE62254.

### Data processing of the scRNA-seq

To preprocess and standardize the tumor specimens from the GSE112302 single-cell sequencing data, the researchers employed the Seurat program available in RStudio. In this study, genes that were expressed in fewer than three and mitochondrial genes that were expressed in more than five percent of the samples were filtered out. Different cell clusters were found using methods for reducing dimension, including principal component analysis (PCA) and t-distributed stochastic neighbor embedding (tSNE). These clusters were then labeled based on marker genes, with a resolution of 0.5. Single cells with more than 7,000 genes were also excluded from further analysis as a means of preventing the formation of possible doublets. Single-cell gene expression data were then adjusted using the "NormalizeData" function using the "LogNormalize" normalization approach. The revised expression matrix served as a data input for further analyses.

### Differentially expressed LMRGs (DE-LMRGs) and enrichment analysis

TCGA RNA-seq data were used to normalize the transcripts per million data. The RNA-seq data were also uniformed with the scale function in the dplyr R package. The sva R package enabled batch correction to be carried out. Differential analysis was then carried out using the collected RNA-seq data. The limma R program screened out the LMRGs and analyzed the differentially expressed genes (DEGs) between normal and malignant tissues. The ggplot2 R tool was then used to display the resultant LMRGs. To gain insights into the potential biological functions and roles of these LMRGs, enrichment analyses were conducted using the "clusterProfiler" R package, utilizing the Gene Ontology (GO) and KEGG databases [32]. To establish a prognostic LMRG signature, the researchers conducted univariate Cox regression analysis to identify LMRGs associated with overall survival. Only LMRGs that demonstrated significant associations below a false discovery rate (FDR) cutoff of 0.05 were chosen for inclusion in the signature.

### Creation and assessment of the LMRG signature

A predictive gene signature was developed using a least absolute shrinkage and selection operator (LASSO) regression model, which helped to select optimal parameters and establish a sequence of genes with predictive capabilities. The implementation of the LASSO methodology through the "glmnet" R package facilitated variable selection and dimensionality reduction, enhancing the accuracy of the model. Risk ratings were assigned to patients based on their gene expression levels, and the prognostic value of the signature was assessed using Kaplan‒Meier plots and receiver operating characteristic (ROC) curves. To validate the robustness of the gene signature model, risk scores were generated using the GSE84437 dataset. ROC analysis was also used to assess the prognostic performance of the model in the validation sets. To find independent prognostic indicators for GC, survival studies were conducted on GEO datasets by means of the "survival" package in R. Both multivariate and univariate analyses were used.

### Construction and validation of the nomogram model

To incorporate all relevant clinicopathological parameters that demonstrated statistical significance, a multivariate Cox analysis was conducted. The resulting findings were utilized to develop a visually informative nomogram model using the "rms" and "survival" R packages. The nomogram model integrated sex, age, T stage, N stage, M stage, grade, clinical stage and risk score, enabling the estimation of patients' 3- and 5-year survival probabilities. Additionally, the ROC curve of the nomogram was plotted to assess its predictive performance in terms of GC patient prognosis.

### Analysis of gene set variation (GSVA)

The "GSVA" R tool was utilized to conduct GSVA on the gene description, allowing us to compare biological processes between groups with moderate and elevated risk scores [33]. The analysis of an expression matrix sample using nonparametric and unsupervised GSVA can reveal alterations in pathways or biological processes. The reference group from the "c2.cp.kegg.v7.1.symbols" category in the molecular signatures database was utilized in this study as the genetic group of interest. The enrichment route was significant if the FDR was less than 0.05.

### Evaluation of the relative distribution of immune cells infiltrating GC tissues

In GC tissues, the composition of tumor-infiltrating immune cells (TIICs) was evaluated using the CIBERSORT deconvolution method within the TCGA GC cohort and corresponding normal samples. The CIBERSORT platform (https://cibersortx.stanford.edu/) was used to obtain the genetic profile matrix for 22 TIICs. By aligning the expression matrix data with the TIIC profile matrix from CIBERSORT, support vector regression was employed to make a percentage matrix representing the proportions of the 22 TIICs in GC tissues, categorized into high- and low-score groups. To ensure the reliability of the results, the Monte Carlo sampling approach was utilized to calculate *P* values for the deconvolution of each sample. The inferred proportions of TIICs assessed by CIBERSORT were considered reliable if the *P* value was below the significance threshold of 0.05. Thus, only samples having a CIBERSORT *P* value of 0.05 were considered appropriate for future study. In addition, the signature matrix was preconfigured with 100 permutations.

### Statistical analyses

R software (version 3.5.1, the R Foundation, Vienna, Austria) was applied to perform the data analysis for this study. One-way analysis of variance (ANOVA) was used for comparisons involving more than two participant groups, while the t test performed by the students was utilized to evaluate differences between two participant sets. The log-rank test was applied to assess group differences, and Kaplan‒Meier analysis was applied to create survival curves. Cox's proportional hazards model was used to find the factors that had a significant impact on survival results. The level of *P* < 0.05 was used to determine two-sided *P* values, and it was deemed significant.

## Results

### Identification of DE-LMRGs in GC specimens and functional enrichment analysis

**Fig. 1:**
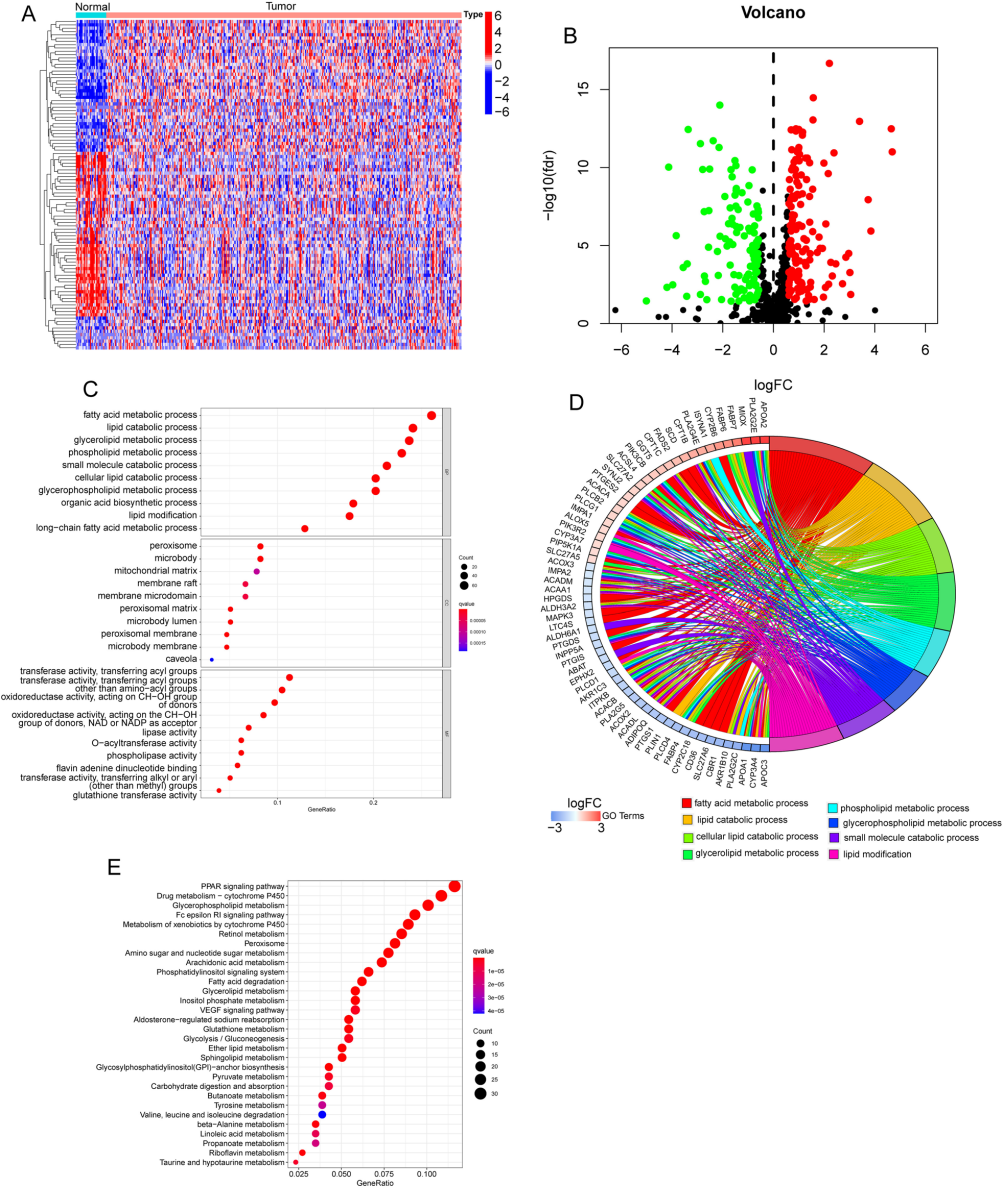
Identification of DE-LMRGs in GC specimens and functional enrichment analysis. **A** and **B** Heatmap and volcano plot of the DE-LMRGs. **C-E** GO and KEGG enrichment analysis for DE-LMRGs

To identify DE-LMRGs between GC specimens and nontumor specimens, we analyzed TCGA datasets using the limma R package. A total of 258 DE-LMRGs were obtained: 107 genes were significantly downregulated, and 151 genes were significantly upregulated (Fig. 1A, B). To examine the functional relevance of DEGs, a Gene Ontology (GO) enrichment analysis was carried out. Several GO keywords showed high enrichment in the context of biological processes, including peroxisome, glycerolipid metabolism, phospholipid metabolism, and fatty acid metabolism. microbody, and transferase activity involving acyl groups (Fig. 1C, D). Furthermore, the KEGG pathway analysis highlighted that 258 DE-LMRGs were prominently associated with various pathways, such as PPAR signaling, drug metabolism-cytochrome P450, glycerophospholipid metabolism, Fc epsilon RI signaling, xenobiotic metabolism by cytochrome P450, retinol metabolism, and peroxisome (Fig. 1E). These findings shed light on the functional roles and potential pathways implicated in the observed gene expression changes.

### Identification of survival-related DE-LMRGs in GC patients

**Fig. 2:**
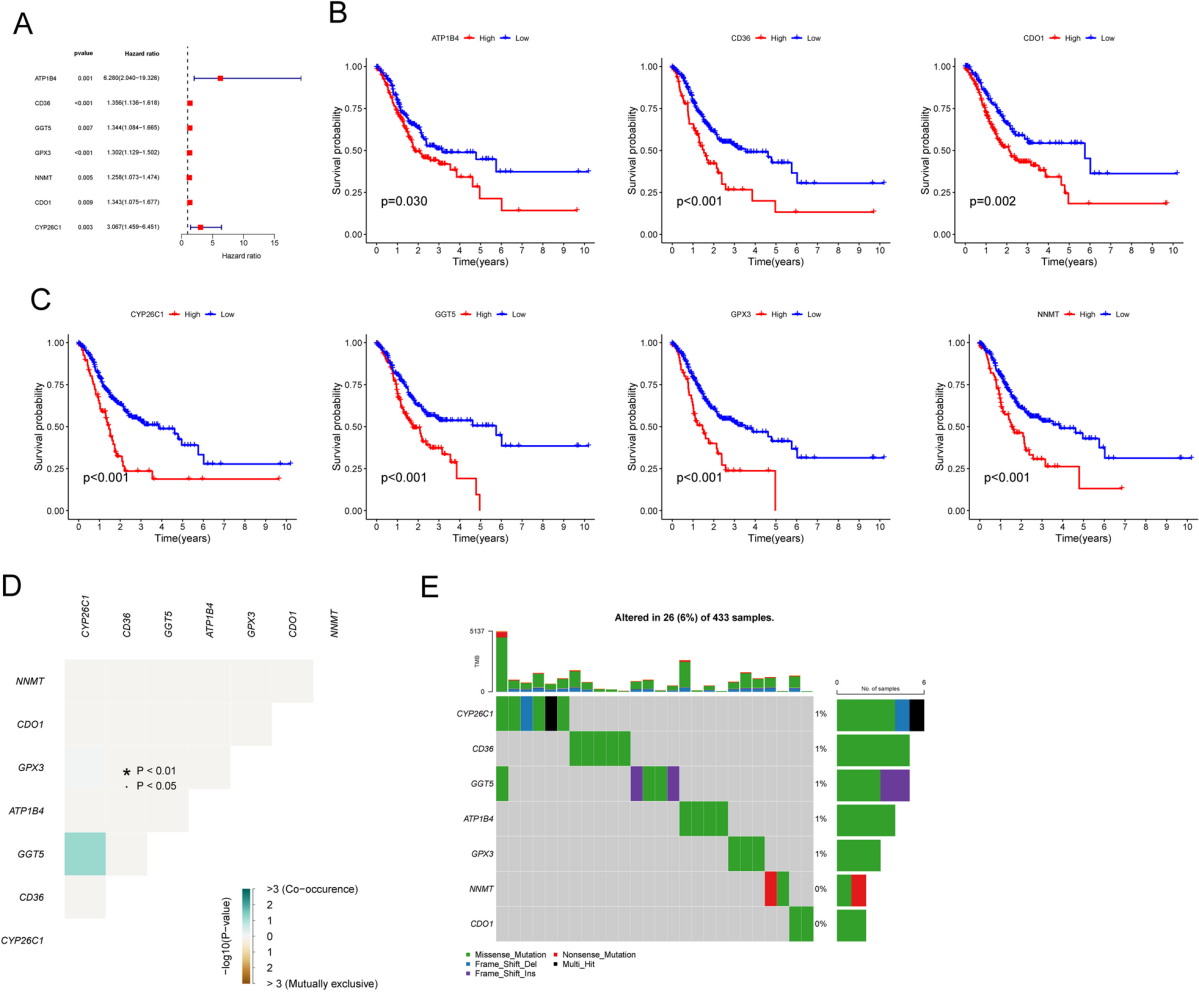
We conducted analyses to identify DE-LMRGs that are linked with GC patient survival. **A** The findings of univariate Cox regression analysis are shown using a forest plot in the TCGA GC cohort, highlighting the relationships between different variables and patient outcomes. Additionally, a Kaplan‒Meier survival analysis was conducted on GC patients to evaluate the prognostic relevance of the seven survival-related DE-LMRGs. **D** and **E** The mutation frequency of seven survival-related DE-LMRGs in 433 patients with GC

Then, we performed univariate analysis to screen survival-related DE-LMRGs in GC patients. As shown in Fig. 2A, we confirmed seven survival-related DE-LMRGs in GC patients, including ATP1B4, CD36, GGT5, GPX3, NNMT, CDO1 and CYP26C1. The Kaplan‒Meier analysis of survival data showed that patients with high expression of all seven of the aforementioned genes had considerably shorter general survival durations than patients with low expression (Figs. 2B, C). To further investigate the association between these genes and survival, the somatic mutation profile of the seven survival-related DE-LMRGs was examined. Among the 433 GC samples analyzed, 26 exhibited mutations in the LMRGs, with a mutation frequency of 6% (Fig. 2D, E). These findings highlight the potential impact of these genes on patient survival and provide insights into the somatic mutation landscape of the DE-LMRGs in GC.

### Cell trajectory analysis identified three GC subsets

**Fig. 3:**
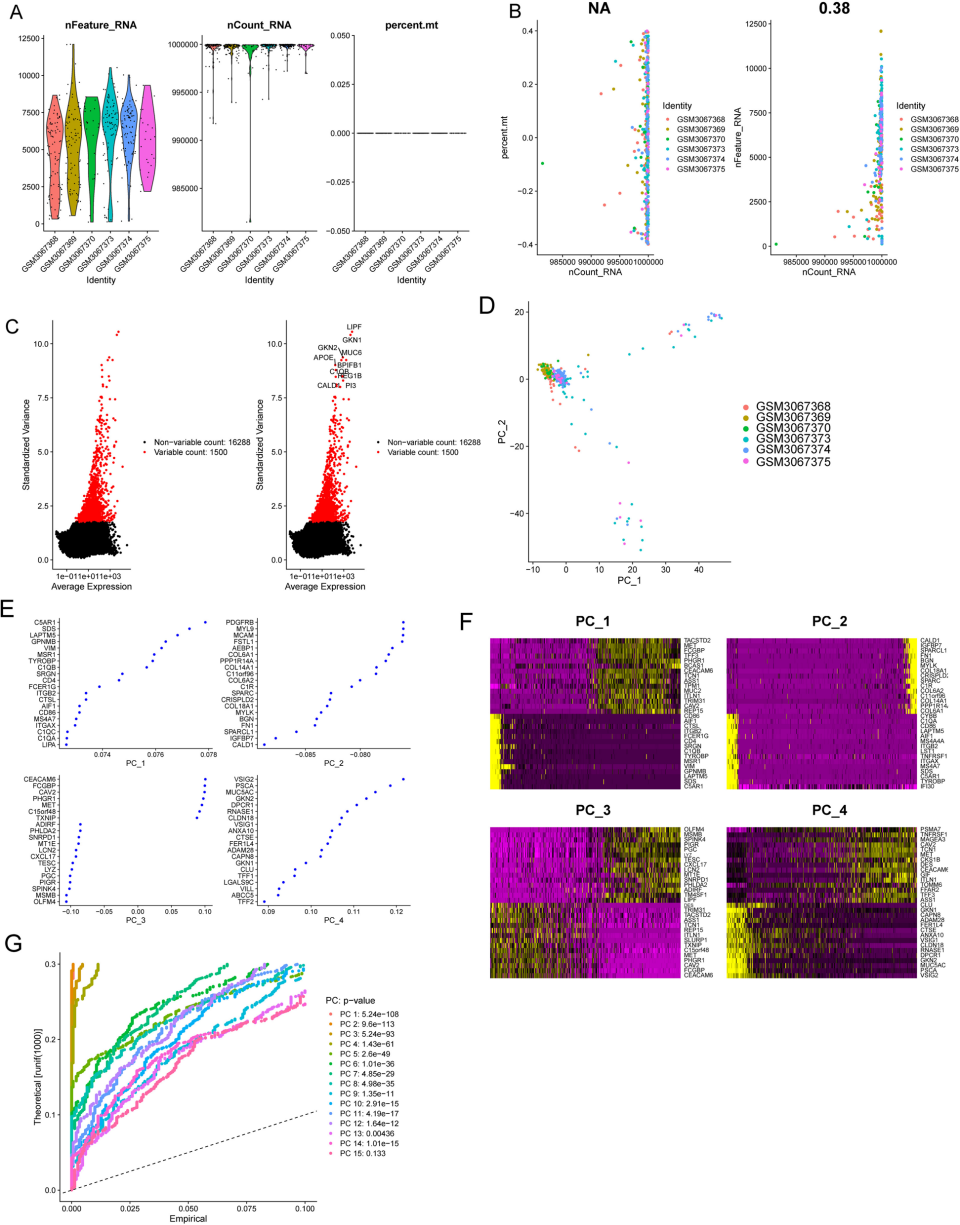
Data normalization and quality assurance for scRNA-seq. **A** A total of 400 cells were tested for further investigation after quality control and normalization removed 2 nonconforming cells. **B** Mitochondrial gene sequences were shown to correlate with sequencing depth. **C** Analysis was performed on a total of 16,288 genes, 14,788 of which showed little change between cells and 1,500 of which showed significant variance. **D** PCA calculated using data from scRNA-seq. **E** and **F** The marker genes and their expression patterns. **G** It was determined that 15 PCs had significant differences when P was less than 0.5

**Fig. 4:**
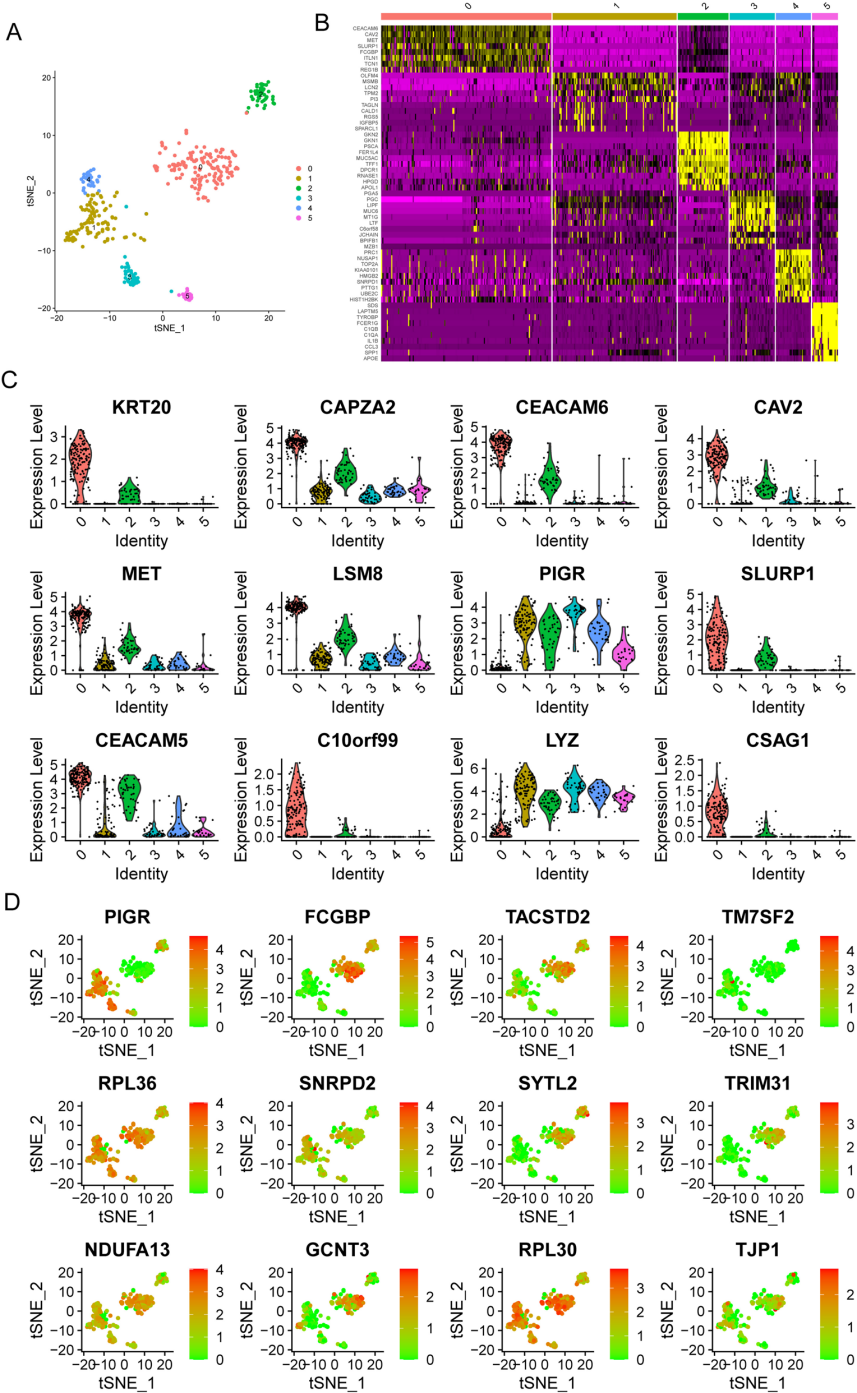
**A** Using the tSNE approach, we classified 400 GC cells into five unique groups. **B **The heatmap shows the highest point 10% of marker genes unique in every group. **C** and **D** Clustering of the vital marker genes by their expression

**Fig. 5:**
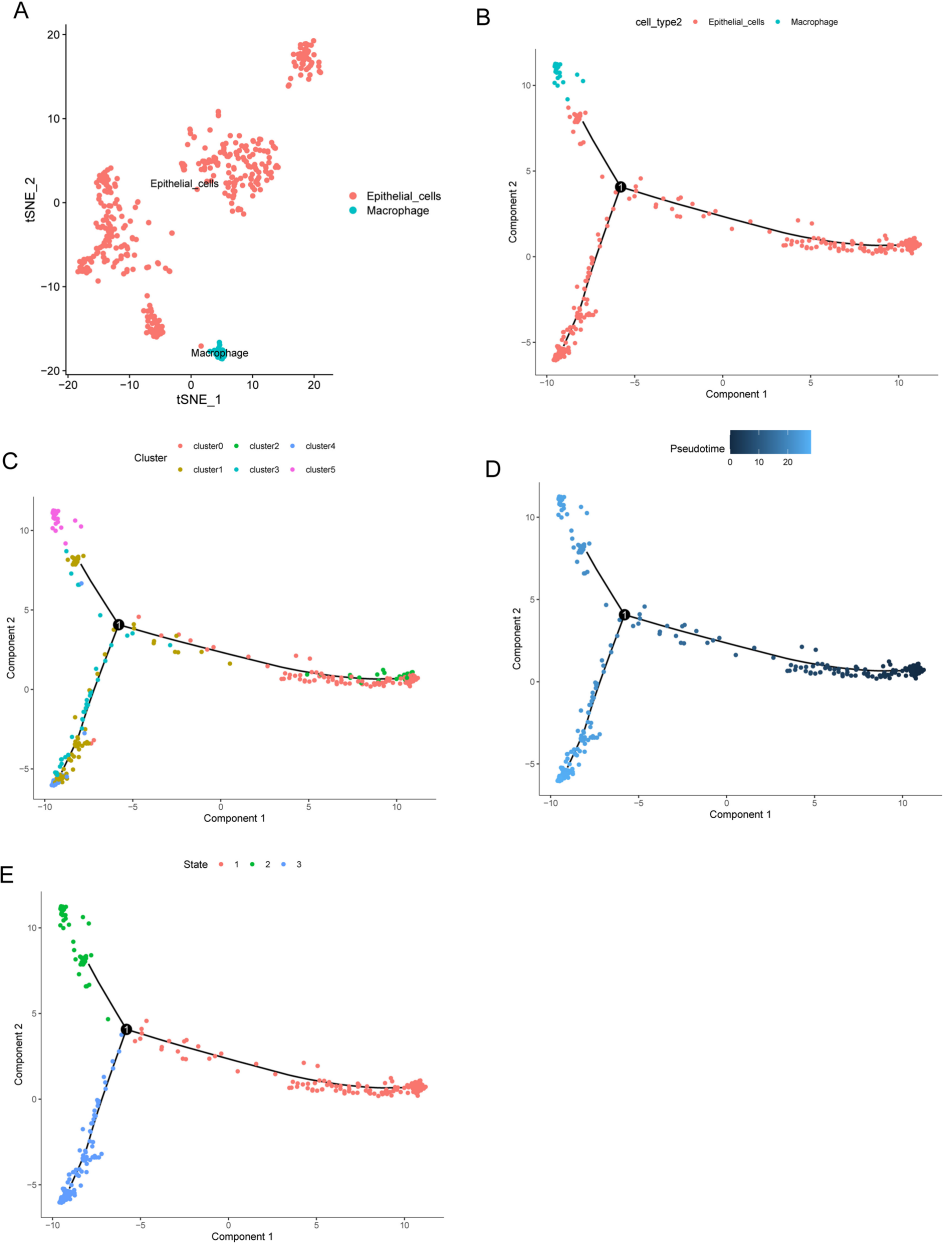
Cell trajectory analysis of GSE112302. **A** Epithelial cells and macrophages are the two types of cells that result from the division of cells. **B-E** Pseudotime and trajectory analysis

The GSE112302 dataset was used to extract 402 cells from 6 different GC sample types for this study. Two cells did not match the defined criteria; hence, 400 cells were chosen for further investigation after rigorous evaluation of quality and normalization (Fig. 3A). These selected cells were deemed suitable for deeper research in line with the study's objectives. There was no link between the sequencing depth and the mitochondrial gene sequences (Fig. 3B). Analysis was performed on a total of 16,288 genes, 14,788 of which showed little change between cells and 1,500 of which showed significant variance (Fig. 3C). The dimensionality of the scRNA-seq data was initially reduced using principal component analysis. The research revealed that no statistically meaningful separation of GC cells was observed (Fig. 3D). The marker genes shown in Fig. 3E displayed unique expression patterns, as shown in Fig. 3F. From these marker genes, 16 principal components (PCs) with major variations were chosen for deeper research (Fig. 3G). The t-distributed stochastic neighbor embedding (tSNE) method was used to divide the 400 GC cells into five groups. The top 10% of the marker genes for each group were depicted in a heatmap following the identification of marker genes by differential analysis (Figs. 4A, B). Figure 4C, D depict the expression profiles of the key marker genes within the five identified clusters. In addition, we categorized the cells based on the flag genes they express, such as epithelial cells and macrophages (Fig. 5A). Then, we performed cell trajectory analysis and identified three branches (Fig. 5B, E). To screen critical genes in GC progression, we analyzed DEGs between the three branches and identified 688 DEGs.

### Construction and assessment of a prognostic model based on LMRGs

**Fig. 6:**
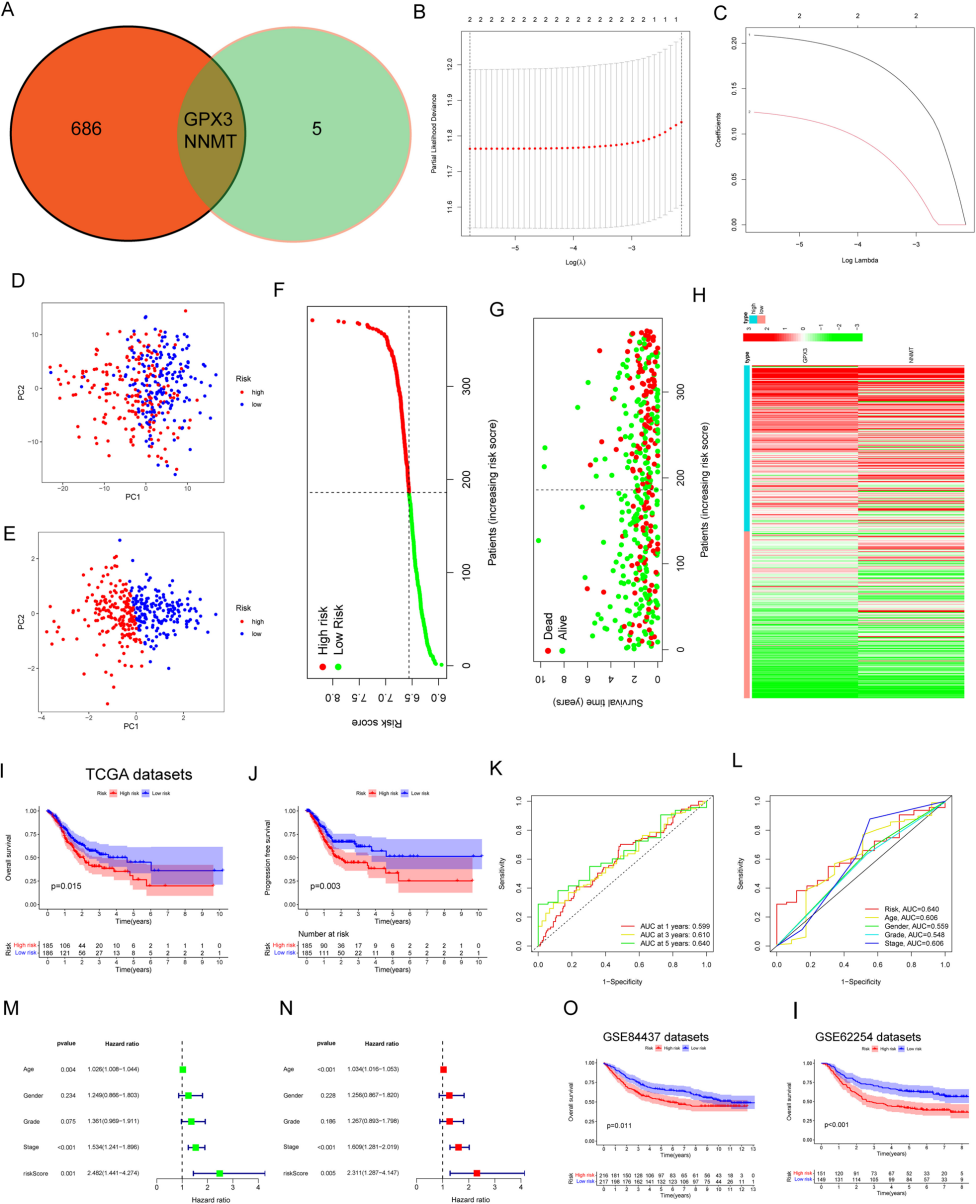
Construction of an LMRG signature and evaluation of the possibility for independent prognostication. **A** Venn diagram to identify the common genes of 688 DEGs between three branches and survival-related DE-LMRGs. **B** and **C** Cvfit and lambda curves demonstrating minimal criterion LASSO regression. **D** and **E** Principal component analysis. **F**–**H** Distributions of overall survival and risk scores, as well as risk score distributions. **I** and **J** Kaplan‒Meier curves for individuals with GC showing their overall survival and survival without progression. **K** The ROC curve demonstrates the innovative model's ability to predict 1-, 2-, and 3-year survival rates in GC patients. **L** The AUC values were obtained to evaluate the risk score's predictive capacity with other prognostic parameters. **M** and **N** The impact of the novel prognostic model on the general survival rate was investigated using results from univariate and multivariate Cox regression analyses. **O** The predictive importance of the newly generated LMRG signature was also validated in the GSE84437 dataset

**Fig. 7:**
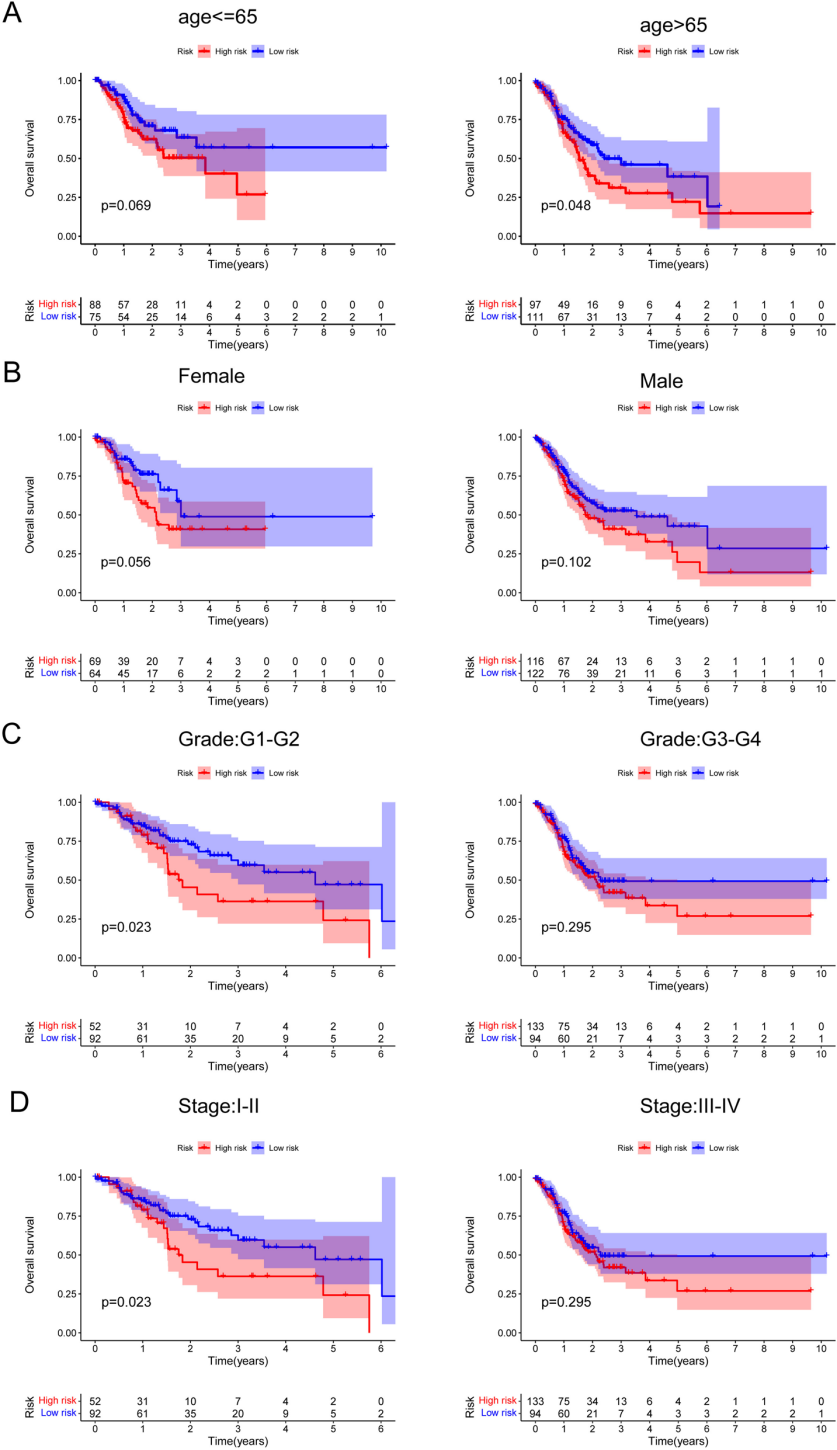
Kaplan‒Meier curves of overall survival differences stratified by (**A**) age, (**B**) sex, (**C**) grade and (**D**) pathological stage between the high-risk groups and low-risk groups

**Fig. 8:**
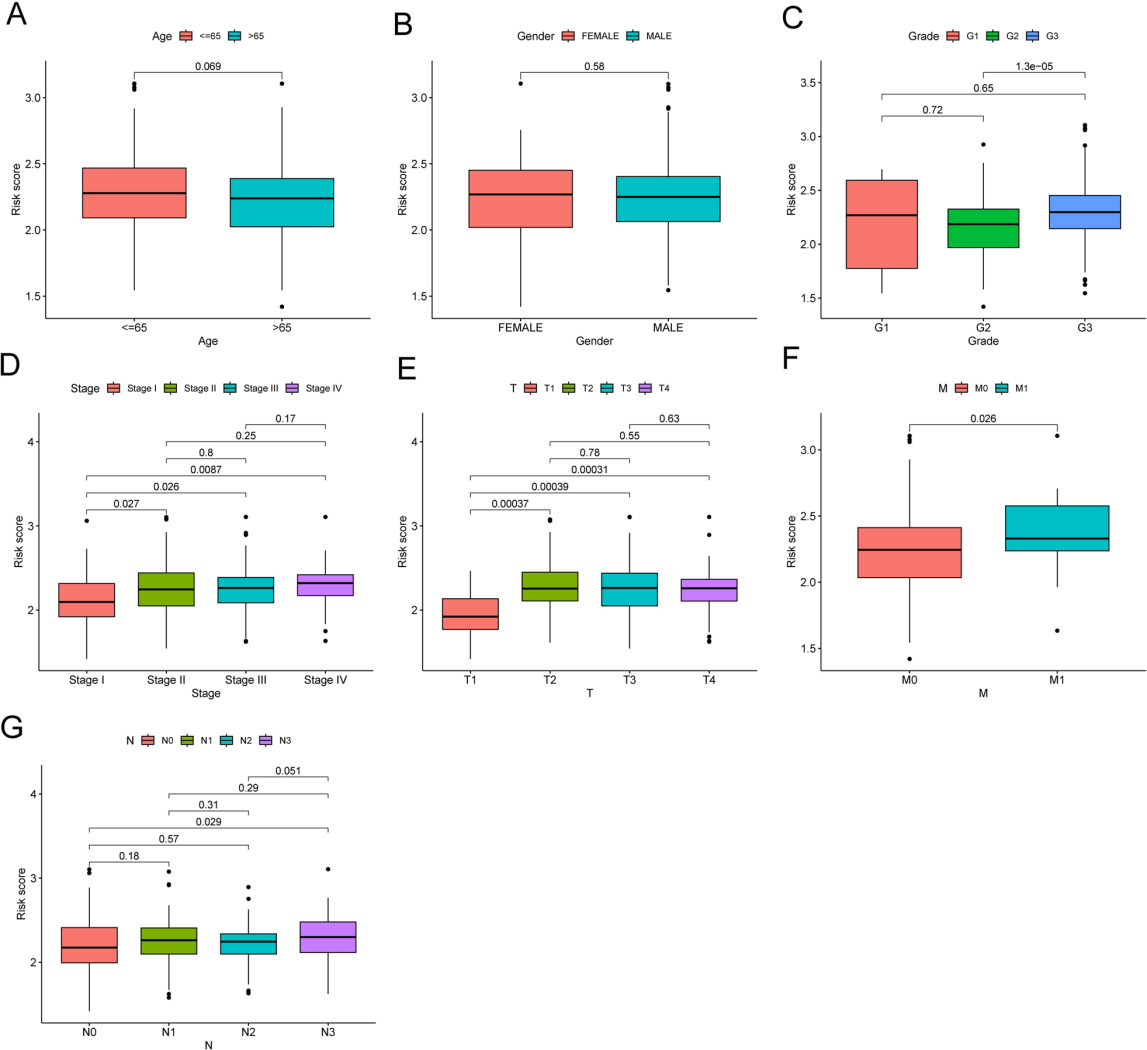
The correlation between clinical characteristics and risk score in patients with GC was examined, including **(A**) age, (**B**) sex, (**C**) grade, (**D**) clinical stage, (**E**) T stage, (**F**) M stage and (**G**) stage

The aforementioned findings led to the identification of 688 differentially expressed genes (DEGs) across the three branches, indicating their potential importance in the development of GC. Subsequently, by intersecting these DEGs, 7 DE-LMRGs associated with patient survival were selected, leading to the identification of 2 crucial genes, GPX3 and NNMT (Fig. 6A). To refine the gene pool further, researchers employed LASSO analysis, which helped reduce the number of genes. Eventually, they established a predictive risk score model by utilizing the variations in two specific genes, GPX3 and NNMT (Fig. 6B, C). Each sample's risk rating was established by considering the following: score of potential danger = 0.20899 * GPX3 + 0.12400 * NNMT. High- and low-risk GC samples were clearly separated using the risk score methodology (Fig. 6D, E). Visualizations of risk score distributions and GC status distributions demonstrated that samples from the aforementioned risk categories were distributed normally (Fig. 6F, H). The examination of Kaplan‒Meier survival data revealed that patients belonging to the group with greater risk exhibited significantly lower rates of general survival (OS) and progression-free survival (PFS), as illustrated in Fig. 6I, J. Figure 6K demonstrates the area under the curve (AUC) values for predicting OS at 1-year, 3-year, and 5-year intervals, which were measured to be 0.599, 0.610, and 0.640, respectively. Furthermore, our findings indicated that the predictive capability of the risk model surpassed that of individual indicators such as age, sex, grade, and stage, as demonstrated in Fig. 6L. These results highlight the potential clinical significance of the risk model based on the identified genes (GPX3 and NNMT), indicating its superiority in predicting patient outcomes compared to traditional single indicators. Cox regression analysis, including both univariate and multivariate approaches, was employed to assess whether the metabolic prognostic signature could predict outcomes autonomously from histological grade, sex, age, and clinical stage. The outcomes of the univariate Cox analysis and multivariate analysis supported the notion that the newly developed prognostic signature could function as an independent prognostic factor (Fig. 6M, N). Furthermore, the prognostic value of the novel prognostic model based on LMRGs was validated using the GSE84437 (Korea) and GSE62254 (Singapore) datasets. As illustrated in Fig. 6O, I, patients classified in the high-risk group demonstrated significantly inferior overall survival compared to those in the low-risk group, thereby strengthening the model's predictive efficacy. Moreover, we performed subgroup analysis according to different clinical features. As shown in Fig. 7A, D, our results suggested that the prognostic model is more suitable for patients with G1-G2 grade GC and those in stages I-II. The performance of the prognostic model was notably improved within these specific subgroups, highlighting its efficacy in predicting outcomes for patients with well to moderately differentiated tumors (G1-G2) and those diagnosed at early stages of the disease (stage I-II). These findings suggest the potential utility of the model for risk assessment and treatment planning within this particular patient cohort. In addition, we performed research on the connection between the risk score and various clinical factors. The findings displayed in Fig. 8A, G revealed a positive link between a higher risk score and advanced grade, clinical stage, T stage, M stage, and N stage. This finding highlights the potential clinical relevance of the risk model in disease forecasting progression and patient prognosis.

### Construction of a nomogram to forecast survival

**Fig. 9:**
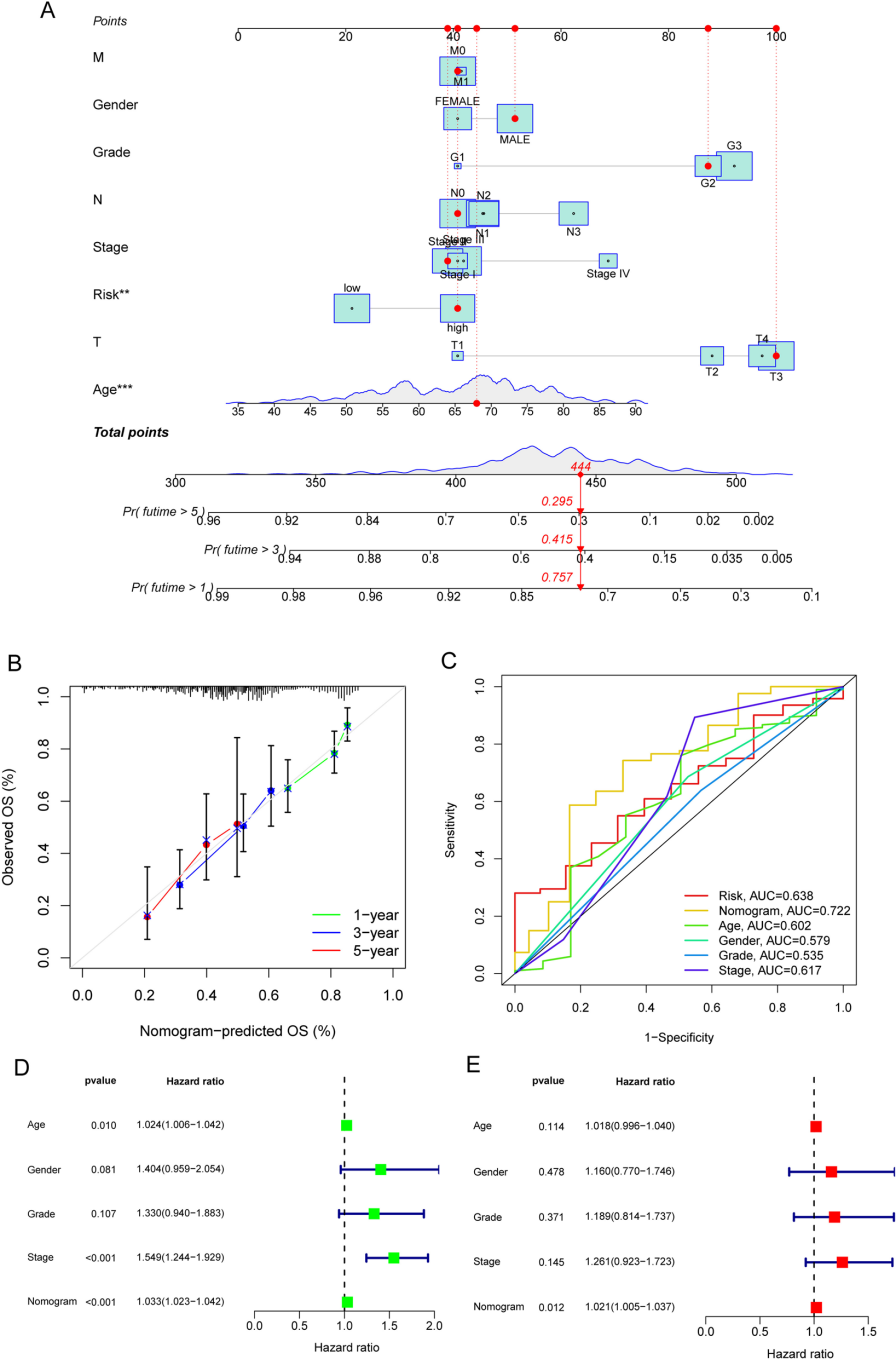
Nomogram to determine the overall chance of people diagnosed with GC surviving the disease. **A** Using the TCGA datasets as input, a prognostic nomogram was developed to estimate patients' chances of surviving GC. **B** Calibration curves of the nomogram for estimating 0.5-, 1-, and 3-year survival using TCGA datasets. **C** ROC curves for evaluating the predictive performance of clinical pathological features and risk score. **D** and **E** The prognostic value of the nomogram was evaluated through the performance of univariate and multivariate Cox regression analyses

To forecast overall survival in GC samples, a nomogram was developed, incorporating age, sex, pathological stage, T stage, N stage, M stage, and a risk score estimate (depicted in Fig. 9A). Calibration curves were generated at 1, 3, and 5 years, confirming the nomogram's ability to predict overall survival in GC patients (Fig. 9B). Furthermore, the results of AUC testing demonstrated that the nomogram (AUC = 0.722) outperformed individual indicators such as age (AUC = 0.602), clinical stage (AUC = 0.617), and the predictive risk assessment model (AUC = 0.638) (Fig. 9C). Multivariate Cox regression analysis further revealed that the novel nomogram serves as an independent predictor of outcome for GC patients (Fig. 9D, E).

### Immune-associated cell and process distribution in the groups with high and low risks

**Fig. 10:**
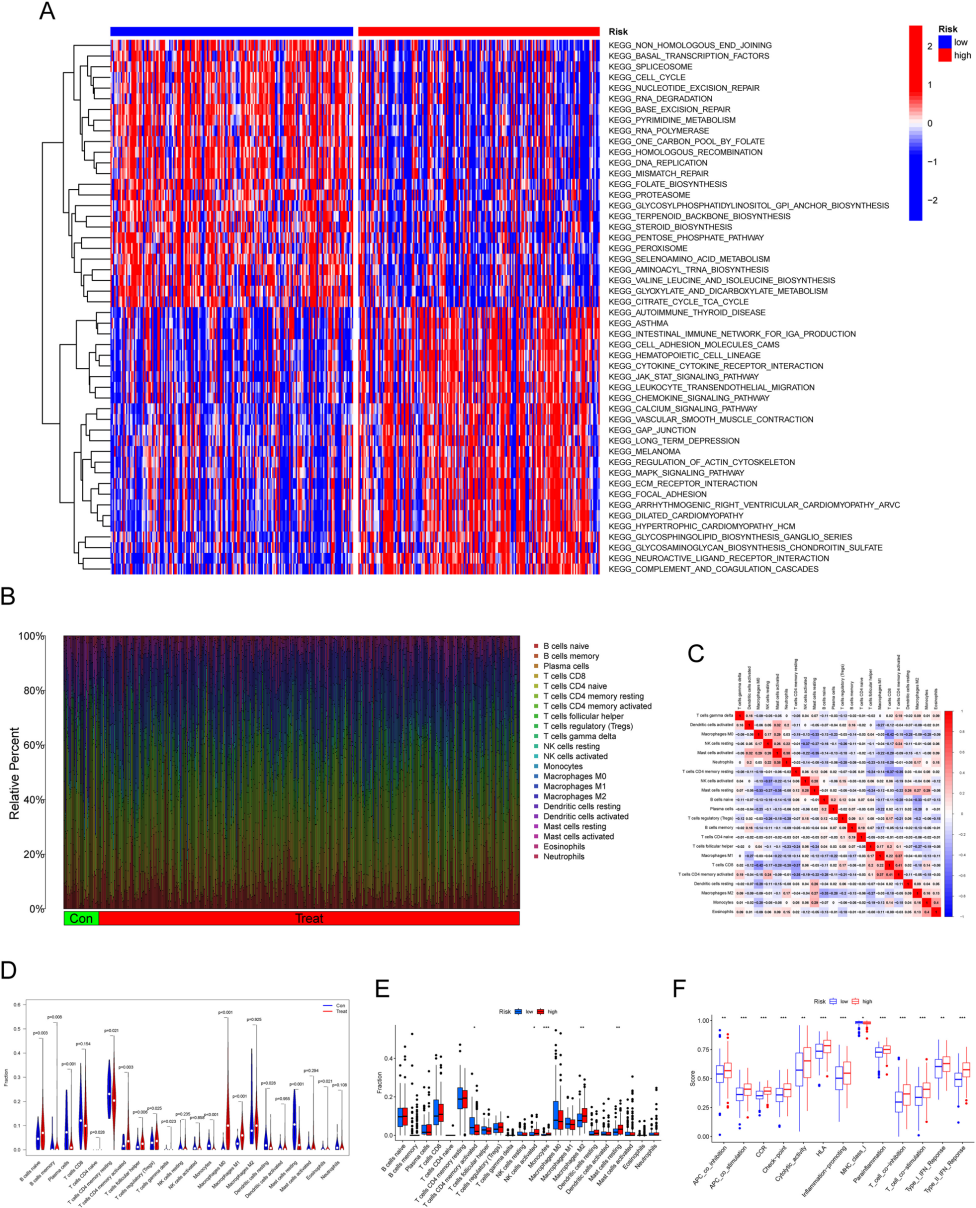
Investigations of immune cell dispersion in GC patients. **A** An illustration of the spatial distribution of immune cell infiltration around patients with GC using a heatmap representation of GSVA enrichment in groups with greater and lower risk. **B** The ratios of 22 immune cell types in normal and GC samples were compared, showing variations in immune cell infiltration. **C** Correlation matrix exhibiting the percentages of all 22 TIIC categories, revealing the makeup and interactions between these immune cells in the GC microenvironment. **D** The percentage of TIICs in GC samples vs control samples was compared using a violin plot. **E** Violin plot comparing TIIC proportions across people with scores with greater and lower scores in GC samples, emphasizing the variations in immune cell composition. **F** Distinct functional profiles related to immune system regulation were observed between patients with greater and lower risk scores, indicating potential variations in immune response and immune-related functions in these individuals

To evaluate the distribution of immune-associated cellular components and biological processes in the high-risk and low-risk groups, we conducted GSVA (Gene Set Variation Analysis) enrichment analysis using gene sets obtained from MSigDB annotated as "c2.cp.kegg.v7.2". The objective of this analysis was to explore the intricate immunological behaviors associated with these two groups. Importantly, our findings revealed immune-related pathways in the low-risk group, suggesting augmented immunoreactivity and potential efficacy of immune responses within this particular subgroup, including KEGG_AUTOIMMUNE_THYROID_DISEASE, KEGG_ASTHMA, KEGG_INTESTINAL_IMMUNE_NETWORK_FOR_IGA_PRODUCTION, KEGG_CELL_ADHESION_MOLECULES_CAMS, and KEGG_HEMATOPOIETIC_CELL_LINEAGE (Fig. 10A). These findings suggest distinct immune cell distributions and processes between groups with greater and lower risks, indicating potential implications for the prognosis and immune status of GC patients. A total of 22 distinct immune cell profiles were developed from GC samples, and the fraction of tumor-infiltrating immune subsets was assessed with the CIBERSORT method to further validate the link between the risk score and TIME (Fig. 10B, C). The levels of several immune cells exhibited a dysregulated level between GC samples and normal samples (Fig. 10D). In the group characterized by a higher risk, there was a substantial enhancement in the activation of memory CD4 T cells. Conversely, in the low-risk group, obvious growth can be seen in the activation of NK cells, as well as an elevation in the presence of activated monocytes, M2 macrophages, and resting mast cells (Fig. 10E). Furthermore, the group at greater risk activated inflammation-promoting function, type II IFN response, type I interferon (IFN) response, T_cell_co_inhibition, and APC_co_inhibition, indicating that activation of the above pathway in patients at greater risk can impact cancer patients undergoing immunotherapy, limiting the effectiveness of immune treatment and promoting tumor evasion from immune attacks (Fig. 10F).

### The expression of GPX3 and NNMT in GC patients, as well as its relationship with clinical variables

**Fig. 11:**
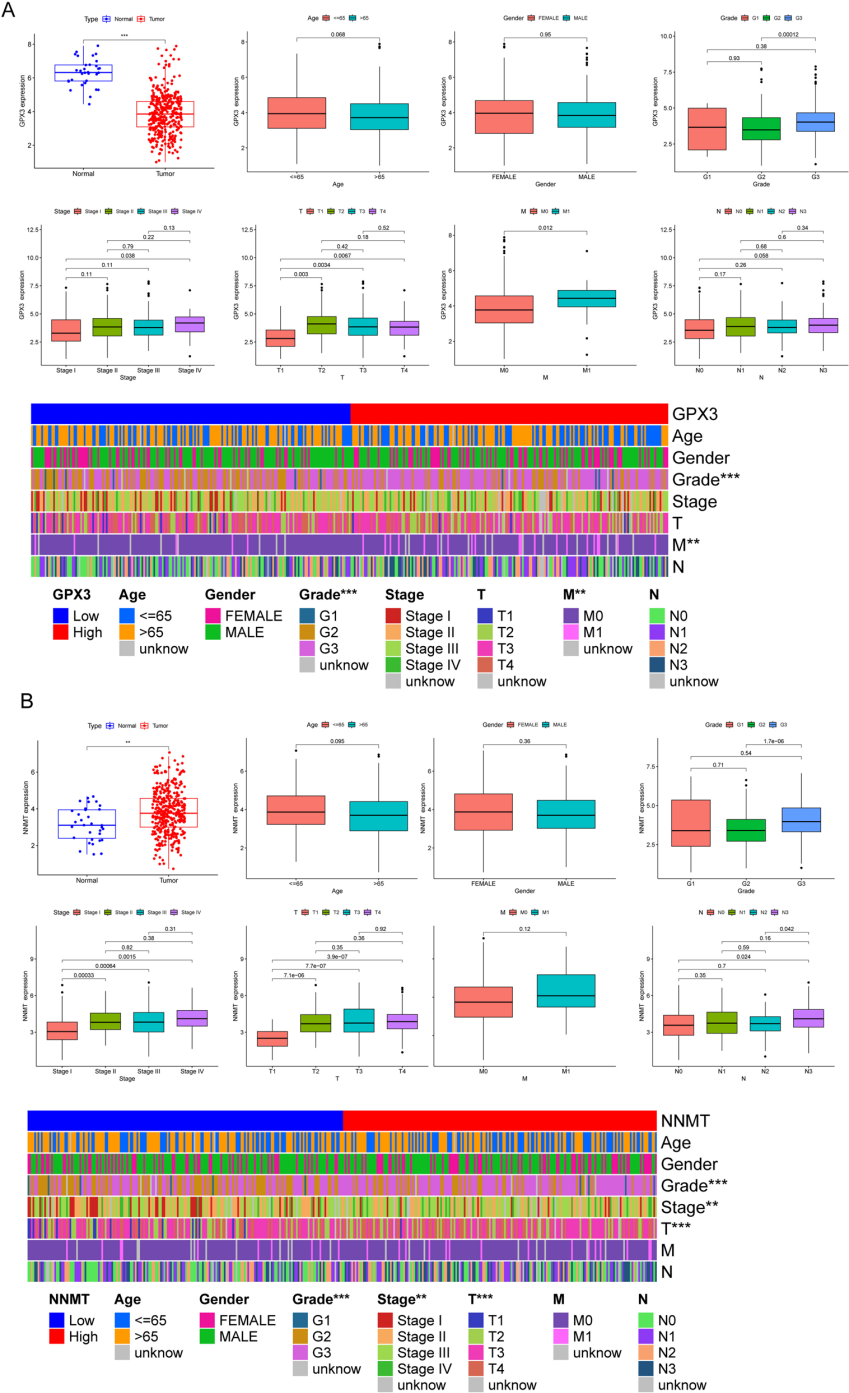
The expression patterns of (**A**) GPX3 and (**B**) NNMT in GC specimens and normal specimens, as well as their association with several clinical factors

Then, using normal and GC specimens from the TCGA databases, we conducted an expression analysis of GPX3 and NNMT. Based on the results shown in Fig. 11A, we found that GPX3 expression was much lower in GC specimens than in nontumor samples, indicating that GPX3 may play a role in the pathogenesis of GC. Additionally, our research found a link between elevated GPX3 expression and more advanced clinical indicators, suggesting a potential function for GPX3 in tumor progression (Fig. 11A). In contrast, NNMT expression was greater in GC specimens than in normal specimens, indicating its potential as a biomarker for GC. Furthermore, our findings indicated that elevated NNMT expression was linked with advanced clinical stage, T stage, N stage, and grade, underscoring its potential relevance as a prognostic indicator (Fig. 11B). These results shed light on the differential expression patterns of GPX3 and NNMT in GC, emphasizing their potential importance in understanding the disease and assessing patient outcomes.

## Discussion

GC is a common malignant tumor, and prognostic prediction is of great significance in guiding treatment strategies and patient management [6, 34]. Advances have been made in the clinical research of GC prognostic prediction methods, including molecular markers, gene expression profiles, clinical pathological factors, imaging features, artificial intelligence and machine learning [35, 36]. However, these methods also have limitations. The diversity and heterogeneity of GC limit the predictive results. Difficulties in data acquisition and processing complicate prognostic prediction. Lack of consistency and standardization make it challenging to compare and apply the results [37, 38]. There are limitations in the accuracy of prediction, and challenges exist in validation and external generalization. Nevertheless, with technological advancements and further research, the accuracy and applicability of these methods are expected to improve, providing better support for personalized treatment of GC patients. Recently, an increasing number of studies have developed novel prognostic models based on different gene types.

Numerous studies suggest that abnormalities in lipid metabolism may be linked to the development and spread of gastrointestinal tract cancers [39, 40]. Lipid metabolism abnormalities conditions increased levels of low-density lipoprotein (LDL), and changes in lipid protein metabolism are examples of lipid metabolism abnormalities [41]. These aberrant conditions could raise the possibility of catching stomach cancer. An elevated risk of stomach cancer is linked to elevated cholesterol levels. Cholesterol is an important component of cell membranes, but high cholesterol levels may have a promoting effect on gastric mucosa and GC [23, 42]. Lipoproteins are proteins responsible for transporting lipids and cholesterol. Lipoprotein abnormalities have been related to an increased risk of stomach cancer, according to research. For instance, an increased possibility of GC may be linked to a higher level of low-density lipoprotein (LDL). Through inflammatory reactions, abnormalities in lipid metabolism may contribute to the growth of stomach cancer. Abnormal lipid metabolism can activate inflammatory cells and release inflammatory factors, thereby exerting an influence on the development of GC [25, 43]. Recent studies have employed LMRGs to create novel predictive models for a variety of tumor types, including papillary thyroid cancer, colon adenocarcinoma, and bladder cancer. In addition, Wei et al. created a risk prediction assessment based on 19 genes associated with lipid metabolism (SMPD3, FUT6, ST3GAL1, B4GALNT1, GALC, CYP1A2, LTC4S, GPX3, GPD2, PIGZ, SMPD2, SGMS1, DPM2, PIK3CA, CDIPT, PLCB3, IPMK, LPL and ACADS). Their model was also confirmed to have a strong ability to predict the clinical prognosis of GC patients [44]. However, their findings were based on the transcriptome. At present, there is currently no information available regarding the prognosis model for LMRGs based on the combined analysis of single-cell genetic sequencing and transcriptome sequencing. In this study, we screened seven survival-related DE-LMRGs in GC patients, including ATP1B4, CD36, GGT5, GPX3, NNMT, CDO1 and CYP26C1. By using scRNA-seq data, we identified 688 genes that may have a significant influence on the differentiation process of GC cells. Importantly, we finally screened two critical genes, GPX3 and NNMT, between the scRNA-seq data and transcriptome data. The GPX3 gene encodes glutathione peroxidase 3, which is an antioxidant enzyme responsible for clearing harmful oxidizing substances such as hydrogen peroxide within cells and maintaining the cellular redox balance [45]. A study found that patients with colorectal cancer have significantly lower levels of GPX3 gene expression than those without the disease. This may be associated with an increase in oxidative stress levels within tumor cells, thereby promoting cancer cell proliferation and survival [46]. A previous study reported that in the HGSA subtype of ovarian cancer, there is a distinct separation between tumors with high and low GPX3 expression. GPX3 is essential for the survival of HGSA ovarian cancer cells in the ascites tumor microenvironment, and it acts as a protective factor against external oxidative stress factors. These results highlight the importance of GPX3 as an adaptive mechanism necessary for transcoelomic metastasis. Zhou et al. reported GPX3 hypermethylation in GC, which indicated a shorter period before the recurrence of the tumor in individuals older than 60 [47]. However, the potential function of GPX3 in GC has not been investigated. Our findings suggested that high GPX3 expression was associated with advanced clinical stage, suggesting that it is a tumor promotor in GC. Reduced histone methylation is a consequence of NNMT's ability to catalyze the methylation of pyridine compounds with S-5′-adenosyl-l-methionine. NNMT is widely expressed in the human body, especially in organs and tissues such as the liver, kidneys, brain, lungs, and adipose tissue, where it is expressed at higher levels [48, 49]. The activity and expression levels of NNMT are regulated by various factors, including genetic variations, environmental factors, and nutritional status. Recent studies have indicated that the NNMT gene is associated with several important physiological and disease processes. For example, the NNMT gene may play a role in energy metabolism, fatty acid oxidation, liver fat accumulation, diabetes, obesity, and cardiovascular diseases [50, 51]. Furthermore, multiple investigations have elucidated a notable correlation between NNMT gene expression and the initiation and advancement of tumors. Significantly, Liang et al. documented an upregulation of NNMT expression in GC and highlighted its role in promoting epithelial-mesenchymal transition (EMT) in GC cells through the activation of TGF-1 expression [52]. These findings collectively suggest that NNMT and GPX3 play pivotal roles as tumor promoters in the progression of GC.

Next, a novel prognostic model incorporating GPX3 and NNMT was developed. Through gene signature analysis, patients with GC were divided into two distinct groups with greater and lower groups. The group with greater risk exhibited significantly lower overall survival rates, with a survival rate of less than 15%, compared to the group with lower risk. This signature's predictive significance was further verified in the GSE84437 (Korea) and GSE62254 (Singapore) datasets. Our findings indicated that the signature's ability to predict or classify certain outcomes, such as disease prognosis or treatment response, has been consistently demonstrated across multiple datasets representing diverse populations. Our results strengthened the confidence in the signature's validity and applicability, as it has shown consistent performance beyond the initial dataset, making it more likely to be a reliable tool for its intended purpose. In addition, the calibration curve of the prognostic nomogram showed that the expected and observed survival rates for each overall survival outcome were in good agreement. After controlling for other clinical parameters, further study suggested that this signature could be a useful prognostic predictor.

The TIME encompasses a complex network of cellular, molecular, and signaling interactions surrounding the tumor [53]. It comprises diverse elements, such as tumor cells, immune cells (including T cells, B cells, and macrophages), blood vessels, stromal cells, cytokines and chemical substances [54]. The TME has a critical influence on the initiation, progression, and therapeutic response of tumors [55]. The inflammatory reactions occurring within the immune microenvironment of the tumor are of significant importance in both the formation and management of tumors. Scientific inquiries have unveiled the significant impact of tumor-associated inflammation on tumor cell proliferation, infiltration, immune cell modulation, and immune checkpoint molecule expression [56, 57]. Within the TME, the presence of immune cells assumes a paramount role in tumor progression and therapeutic interventions. Extensive studies have established a robust association between the abundance and functional attributes of immune cell populations, such as tumor-infiltrating lymphocytes and tumor-associated macrophages, and patient prognosis [58, 59]. Hence, modulating the activity and functionality of tumor-associated immune cells may become a novel therapeutic strategy. The process of vascularization within the TIME is necessary in the development and spread of tumors. Recent research indicates that inhibiting tumor-associated angiogenesis may enhance the efficacy of immunotherapy by reducing tumor oxygen and nutrient supply and improving immune cell infiltration and activity. According to a recent study, we identified a noteworthy enrichment of monocytes, M2 macrophages, and resting mast cells in the high-risk group, indicating a potential association with increased risk. M2 macrophages are a subpopulation of macrophages that function primarily in immune regulation, tissue repair, and immune tolerance. In certain circumstances, an increase in M2 macrophages may be associated with tumor progression and unfavorable prognosis. M2 macrophages can suppress immune responses and promote tumor evasion of immune surveillance. They release anti-inflammatory factors and immunosuppressive molecules that inhibit the activity of other immune cells, hindering immune attack against tumors. In addition, M2 macrophages can promote tumor angiogenesis (blood vessel formation), providing the tumor with the necessary oxygen and nutrients, thereby facilitating tumor growth and metastasis. Furthermore, we noted the activation of pathways related to inflammation promotion, type II interferon (IFN) response, type I IFN response, T_cell_co-inhibition, and APC_co-inhibition in the high-risk group. This observation suggests a potential involvement of these pathways in driving the increased risk associated with this particular group. These findings suggest that activating these pathways in patients living in dangerous situations may have detrimental effects on the efficacy of immunotherapy and facilitate tumor immune evasion. In summary, our results indicate that patients with a higher risk profile in GC may exhibit reduced responsiveness to immunotherapy.

### Study strengths and limitations

We first performed integrated analysis of single-cell and bulk RNA sequencing data to develop a novel prognostic model for gastric cancer. Nevertheless, several limitations should be considered in our study. First, the primary data source utilized was predominantly derived from the TCGA database, which predominantly represents individuals of European or Asian descent. Extreme care is warranted when extrapolating our results to patients of different races. Second, a tumor prognosis model requires a large amount of clinical data for training and validation. When the sample size is within a scope, it can potentially affect the performance and reliability of the model. This can lead to insufficient predictive capability or the problem of overfitting. Third, the potential functions of NNMT and GPX3 were not investigated in this study.

## Conclusion

In summary, we used two LMRGs to create a predictive signature for GC patient outcomes. In addition to providing a possible treatment target for GC patients, this signature may be used to better understand the mechanics of lipid metabolism and provide more accurate, individual prognosis predictions.

## Data Availability

The datasets used and/or analyzed during the current study are available from the corresponding author upon reasonable request.
